# Supplementary material for: Multipotent Cholinesterase Inhibitors for the Treatment of Alzheimer’s Disease: Synthesis, Biological Analysis and Molecular Docking Study of Benzimidazole-Based Thiazole Derivatives
Source: Molecules. 2022 Sep 18;27(18):6087. doi: 10.3390/molecules27186087 (PMC9504419; doi:10.3390/molecules27186087)
Supplement: Supplementary file 1 [file molecules-27-06087-s001.zip › molecules-1880506-supplementary.pdf]

# Multipotent cholinesterase inhibitors for the treatment of Alzheimer's disease: Synthesis, biological analysis and molecular docking study of benzimidazole based thiazole derivatives

**Rafaqat Hussain[1], Hayat Ullah [2,*], Fazal Rahim[1,*], Maliha Sarfraz[3], Muhammad Taha[4], Rashid Iqbal[5], Wajid Rehman[1], Shoaib Khan[1], Syed Adnan Ali Shah[6,7], Sajjad Hyder[8], Majid Alhomrani [9,10], Abdulhakeem S. Alamri [9,10], Osama Abdulaziz [9], Mahmoud A. Abdelaziz [11]**

1 Department of Chemistry, Hazara University, Mansehra-21120, Pakistan.
2 Department of Chemistry, University of Okara, Okara-56300, Punjab, Pakistan
4 Department of Zoology, Wildlife and Fisheries, University of Agriculture Faisalabad, 38000, Pakistan
3 Department of Clinical Pharmacy, Institute for Research and Medical Consultations (IRMC), Imam Abdulrahman Bin Faisal University, P.O. Box 1982, Dammam 31441, Saudi Arabia
5 Department of Agronomy, Faculty of Agriculture and Environment, The Islamia University of Bahawalpur Pakistan
6 Atta-ur-Rahman Institute for Natural Product Discovery (AuRIns), Universiti Teknologi MARA Cawangan Selangor Kampus Puncak Alam, Bandar Puncak Alam, Selangor 42300, Malaysia
7 Faculty of Pharmacy, Universiti Teknologi MARA Cawangan Selangor Kampus Puncak Alam, Bandar Puncak Alam, Selangor 42300, Malaysia
8 Government College Women University Sialkot, Pakistan
9 Department of Clinical Laboratories Sciences, The faculty of Applied Medical Sciences, Taif University, Taif, Saudi Arabia.
10 Centre of Biomedical Sciences Research (CBSR), Deanship of Scientific Research, Taif University, Saudi Arabia
11 Department of Chemistry, Faculty of Science, University of Tabuk, P. O. Box 741, Tabuk-71491, Kingdom of Saudi Arabia
* Correspondence: ayaanwazir366@gmail.com(H.U.); fazalstar@gmail.com (F.R.)

**Compound-24 against acetylcholinesterase (AChE)**

**3D**

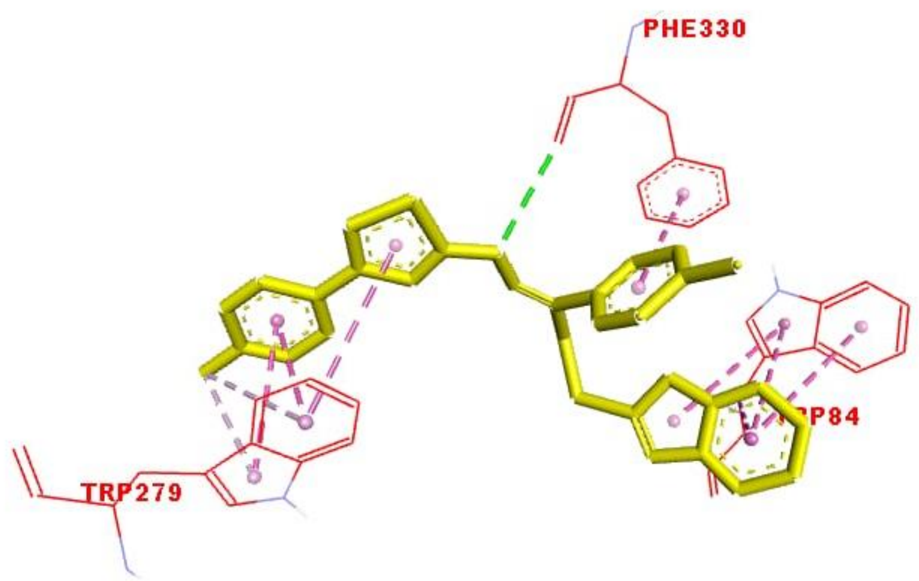


**2D**

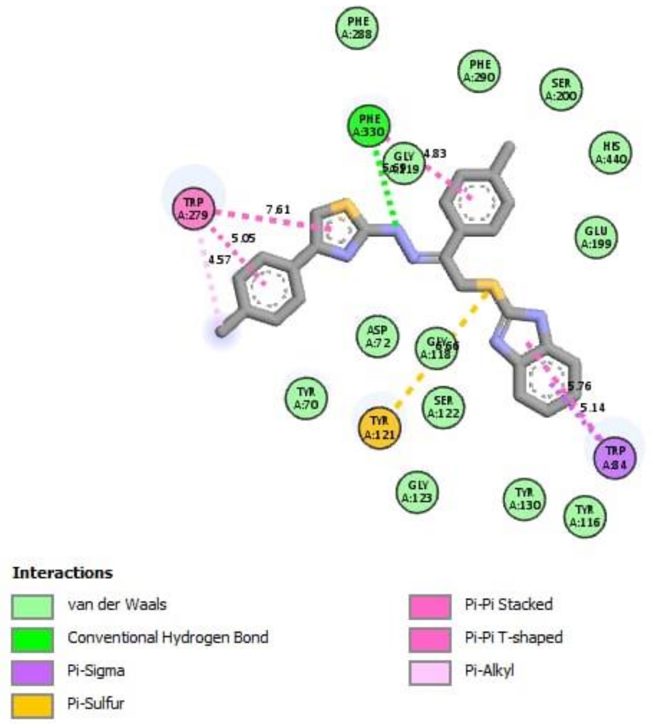


**Figure S1:**Protein Ligand interaction (PLI) profile of compounds **24** against acetylcholinesterase (AChE).

<table>
<tr><th colspan="2">Compound-24 against butyrylcholinesterase (BuChE)</th></tr>
<tr><td>3D</td><td>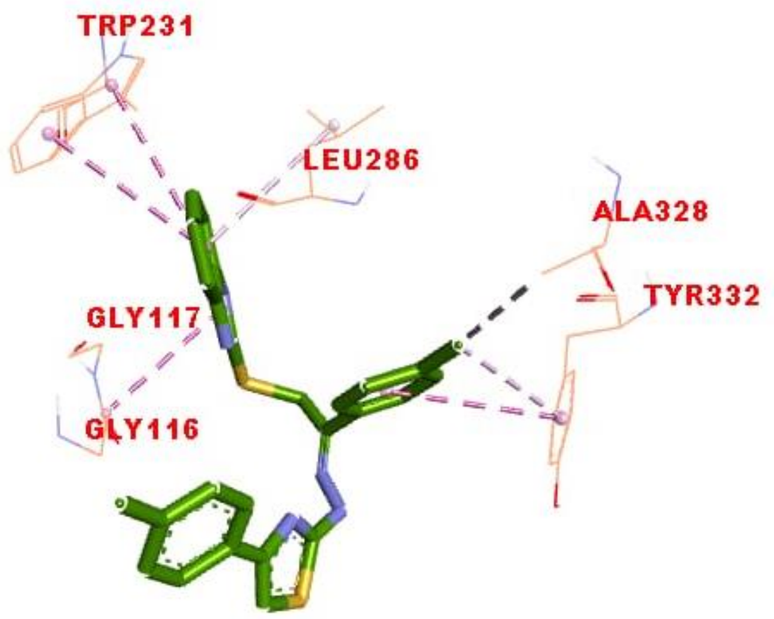
</td></tr>
<tr><td>2D</td><td>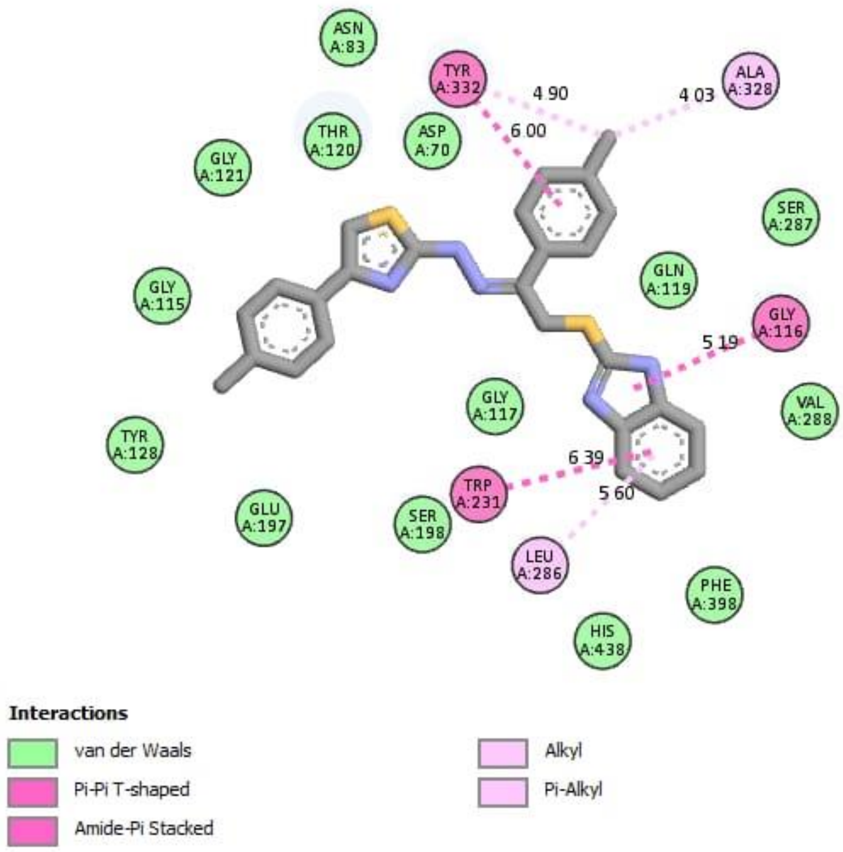
</td></tr>
</table>

**Figure S2:**Protein Ligand interaction (PLI) profile of compounds **24** against butyrylcholinesterase (BuChE).

| Compound-7 against acetylcholinesterase (AChE) | |
|---|---|
| 3D | 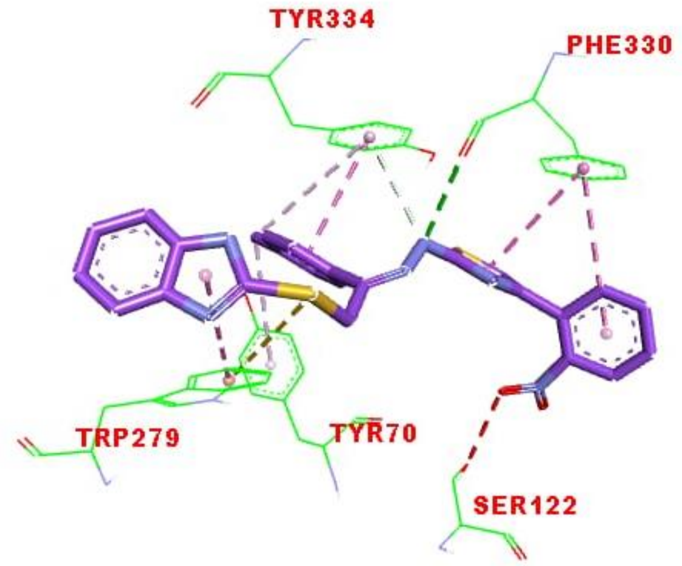 |
| 2D | 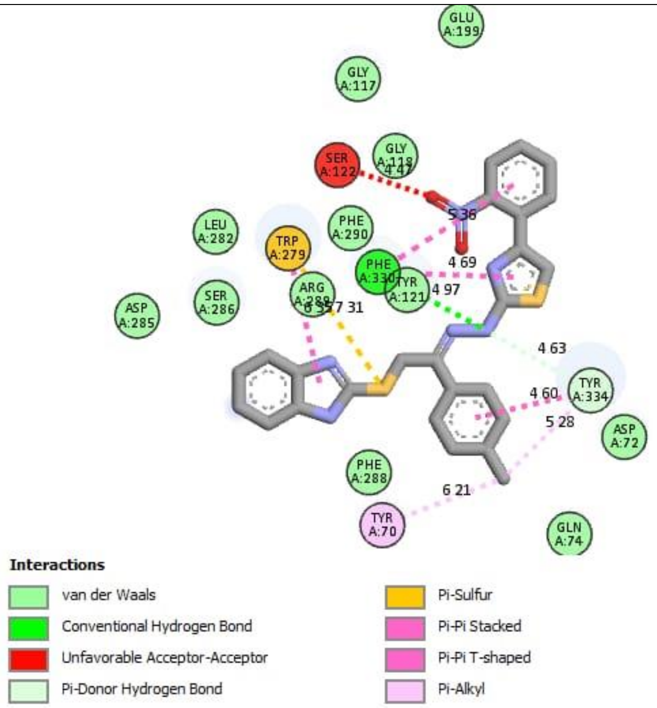 |





**Figure S3:** Protein Ligand interaction (PLI) profile of compounds **7** against acetylcholinesterase (AChE).

| Compound-7 against butyrylcholinesterase (BuChE) | |
|---|---|
| 3D | 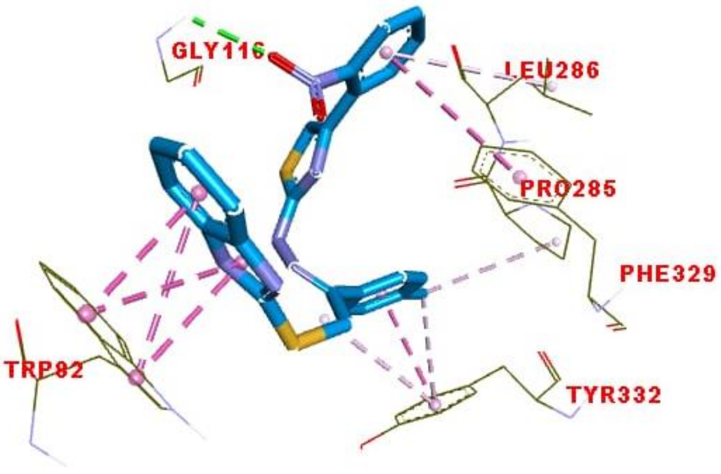 |
| 2D | 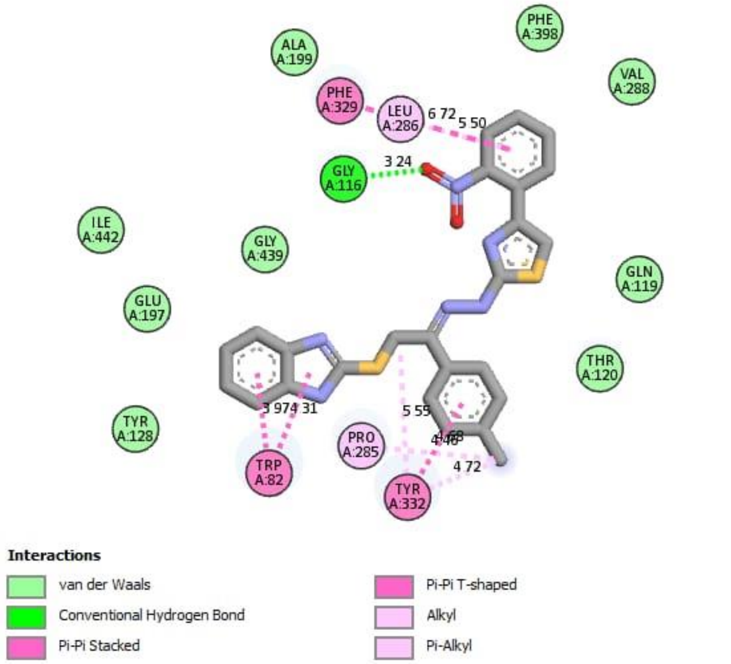 |





**Figure S4:** Protein Ligand interaction (PLI) profile of compounds **7** against butyrylcholinesterase (BuChE).

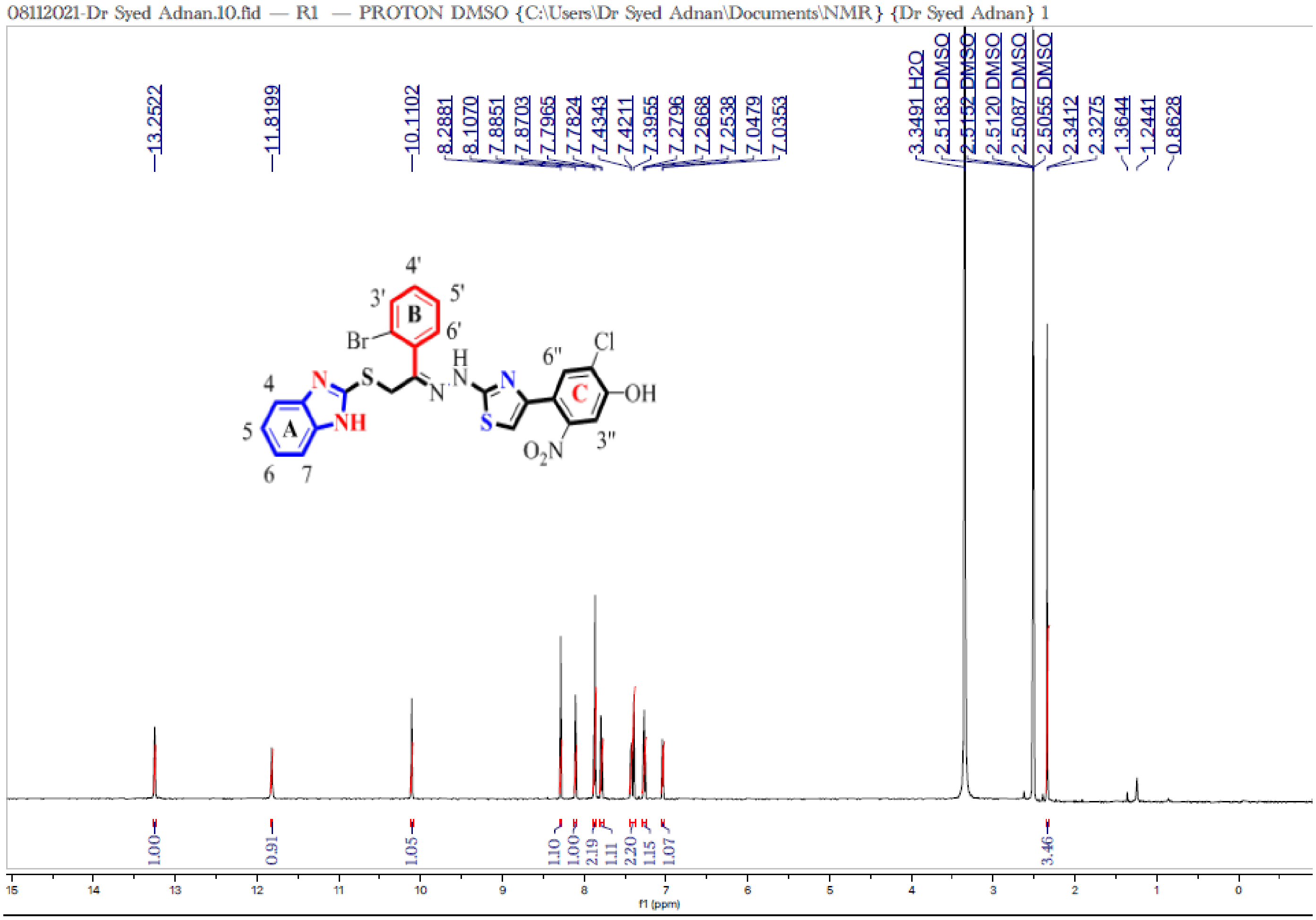


**Figure S5:** $^{1}$H-NMR of (*E*)-4-(2-(2-(2-((1H-benzo[d]imidazol-2-yl)thio)-1-(2-bromophenyl)ethylidene)hydrazinyl)thiazol-4-yl)-2-chloro-5-nitrophenol (1)

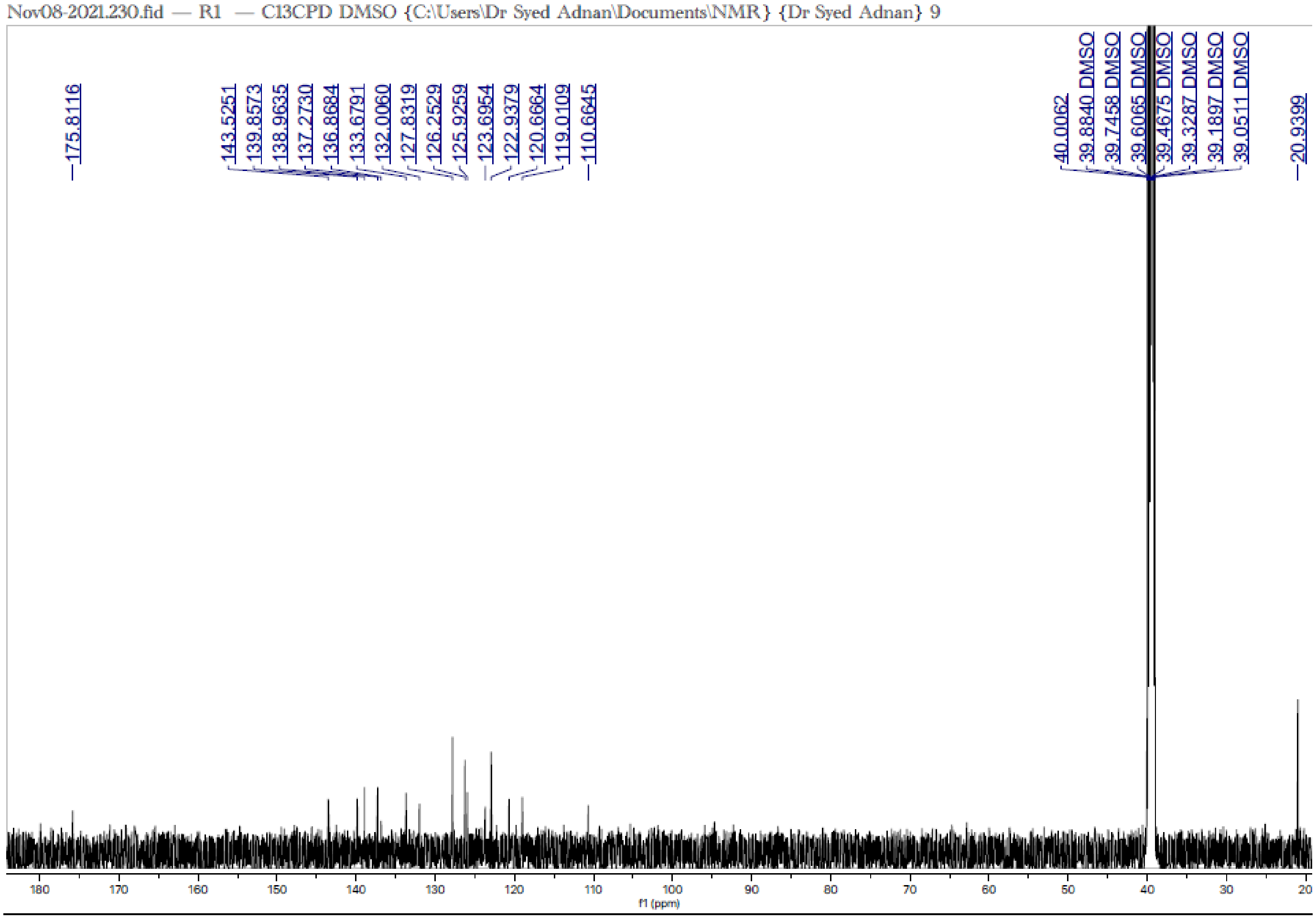


**Figure S6:** $^{13}$C-NMR of (*E*)-4-(2-(2-(2-((1H-benzo[d]imidazol-2-yl)thio)-1-(2-bromophenyl)ethylidene)hydrazinyl)thiazol-4-yl)-2-chloro-5-nitrophenol (1)

08112021-Dr Syed Adnan.20.fid — R2 — PROTON DMSO {C:\Users\Dr Syed Adnan\Documents\NMR} {Dr Syed Adnan} 2

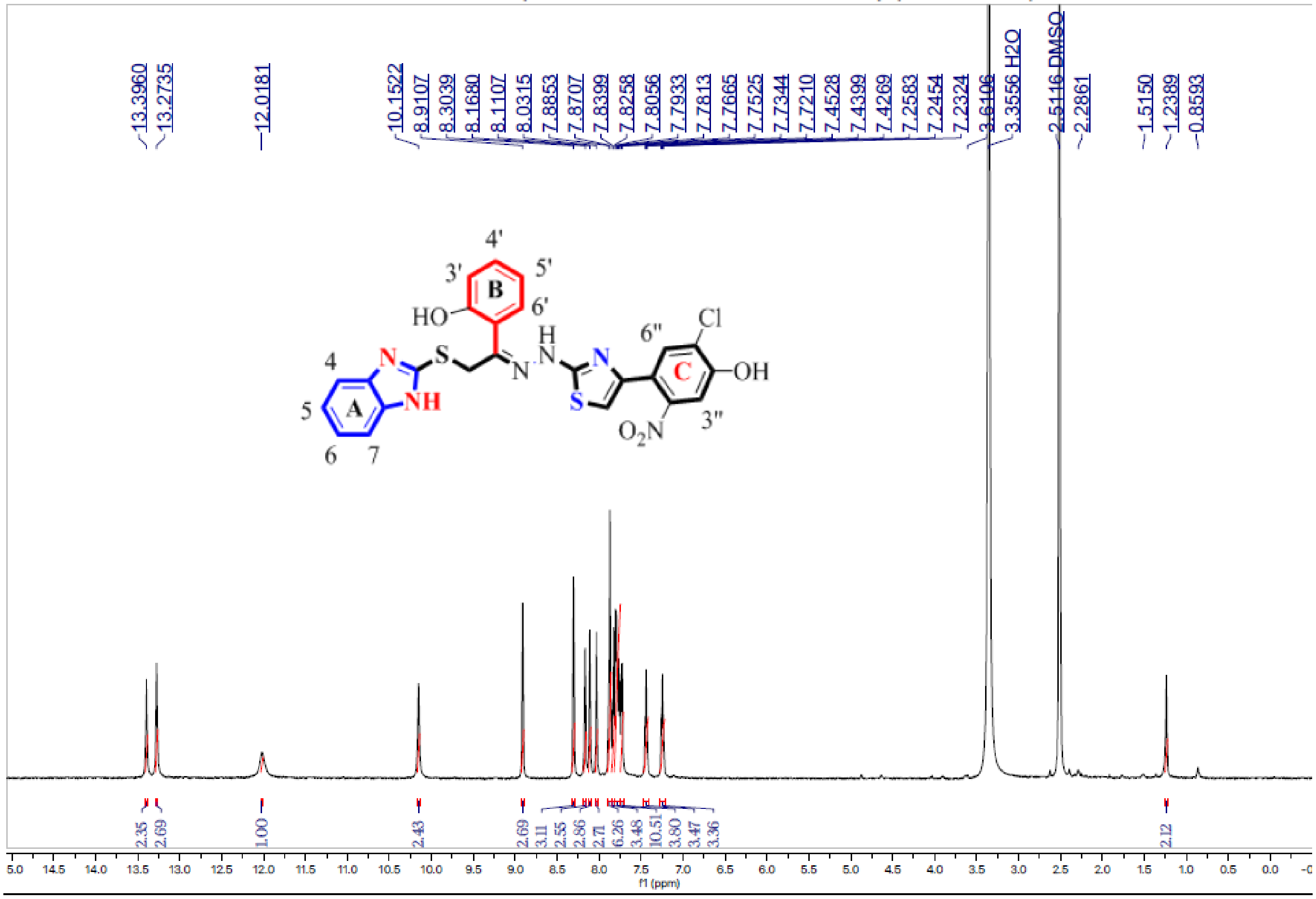

**Figure S7:** ¹H-NMR of (E)-4-(2-(2-(2-((1H-benzo[d]imidazol-2-yl)thio)-1-(2-hydroxyphenyl)ethylidene)hydrazinyl)thiazol-4-yl)-2-chloro-5-nitrophenol (2)

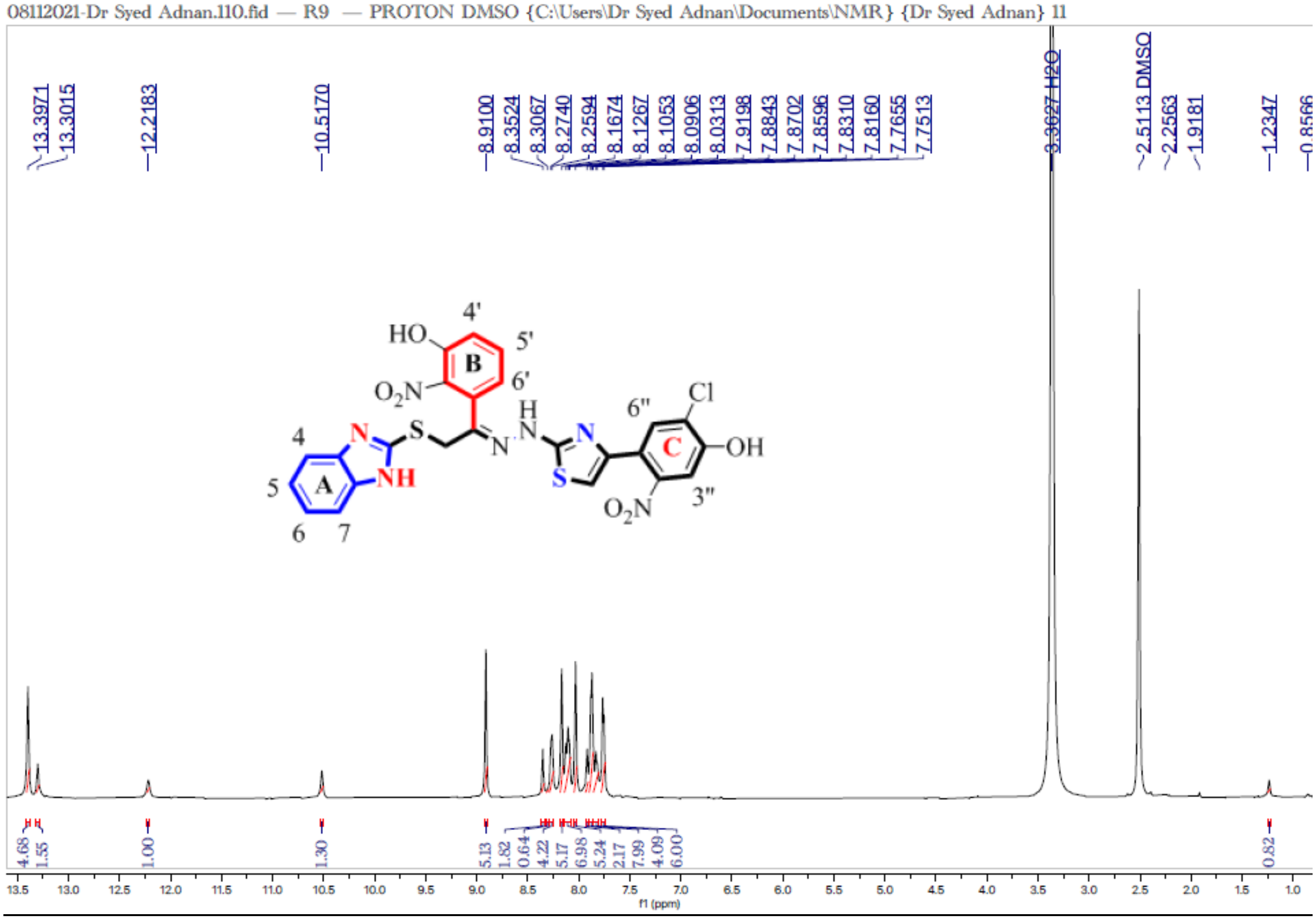


**Figure S8:** $^{1}$H-NMR of (E)-4-(2-(2-(2-((1H-benzo[d]imidazol-2-yl)thio)-1-(3-hydroxy-2-nitrophenyl)ethylidene)hydrazinyl)thiazol-4-yl)-2-chloro-5-nitrophenol (9)

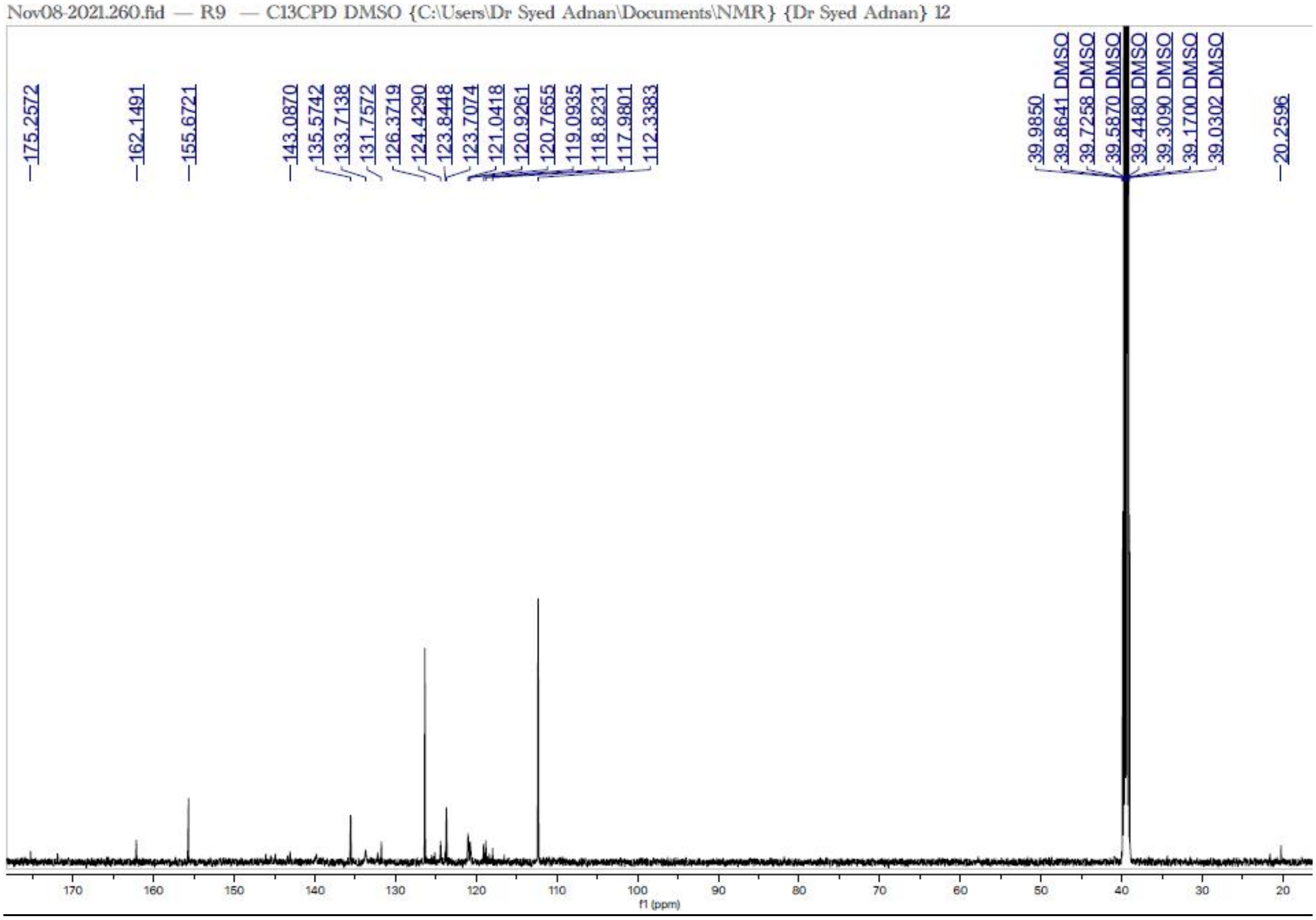


**Figure S9:** $^{13}$C-NMR of (E)-4-(2-(2-(2-((1H-benzo[d]imidazol-2-yl)thio)-1-(3-hydroxy-2-nitrophenyl)ethylidene)hydrazinyl)thiazol-4-yl)-2-chloro-5-nitrophenol (9)

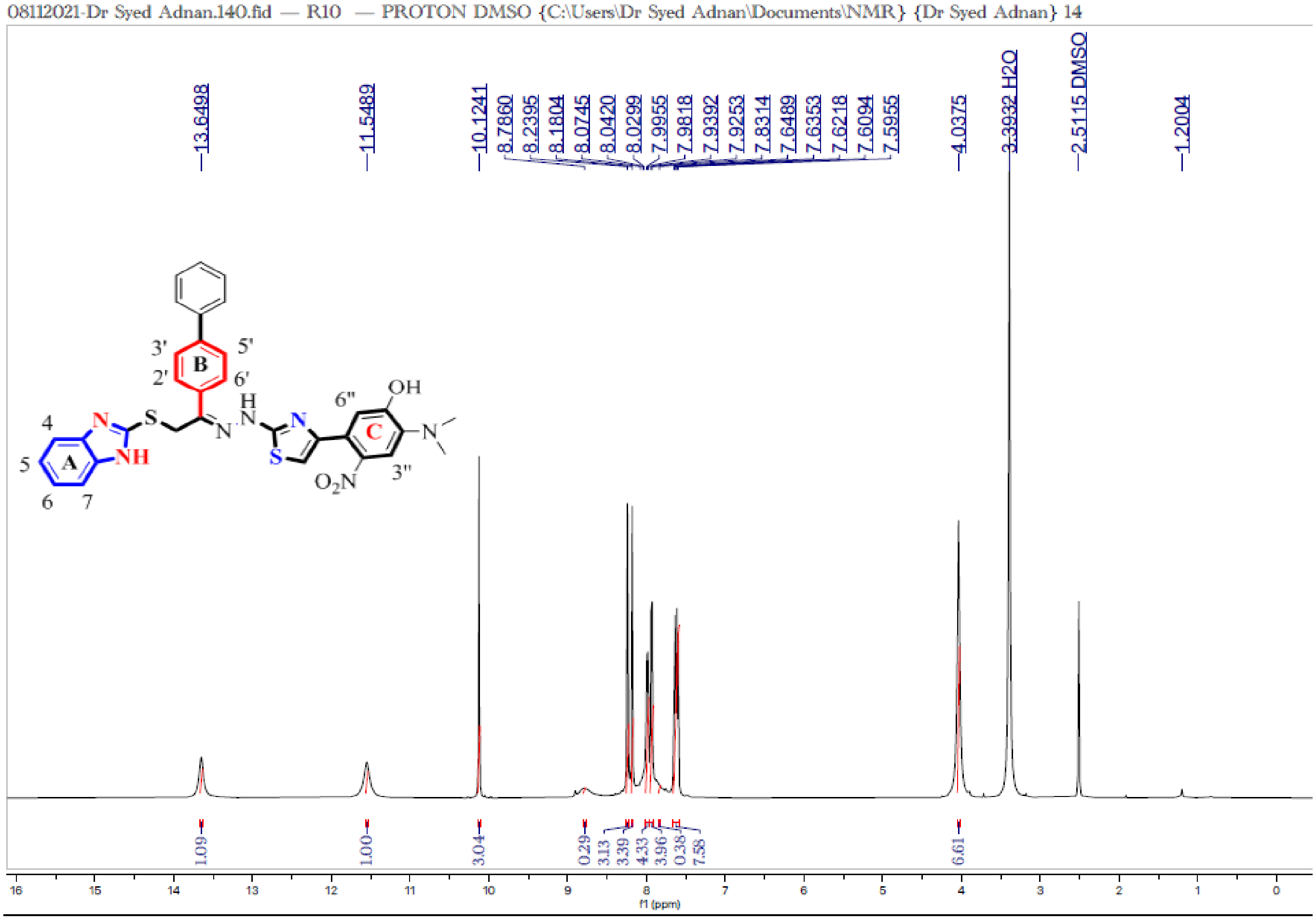


**Figure S10:** $^1$H-NMR (E)-5-(2-(2-(2-((1H-benzo[d]imidazol-2-yl)thio)-1-([1,1'-biphenyl]-4-yl)ethylidene)hydrazinyl)thiazol-4-yl)-2-(dimethylamino)-4-nitrophenol (10)

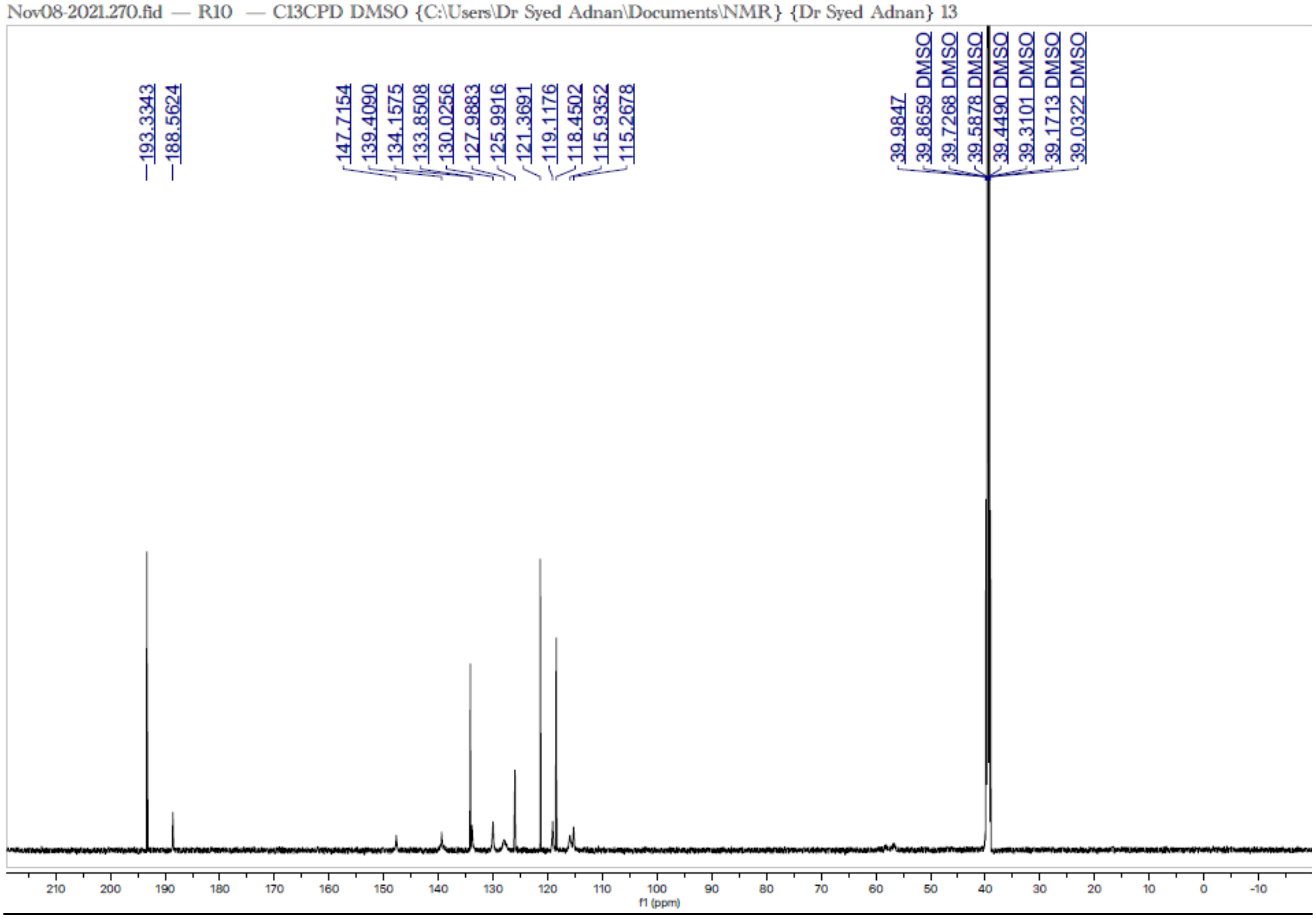


**Figure S11:** $^{13}$C-NMR (E)-5-(2-(2-(2-((1H-benzo[d]imidazol-2-yl)thio)-1-([1,1'-biphenyl]-4-yl)ethylidene)hydrazinyl)thiazol-4-yl)-2-(dimethylamino)-4-nitrophenol (10)

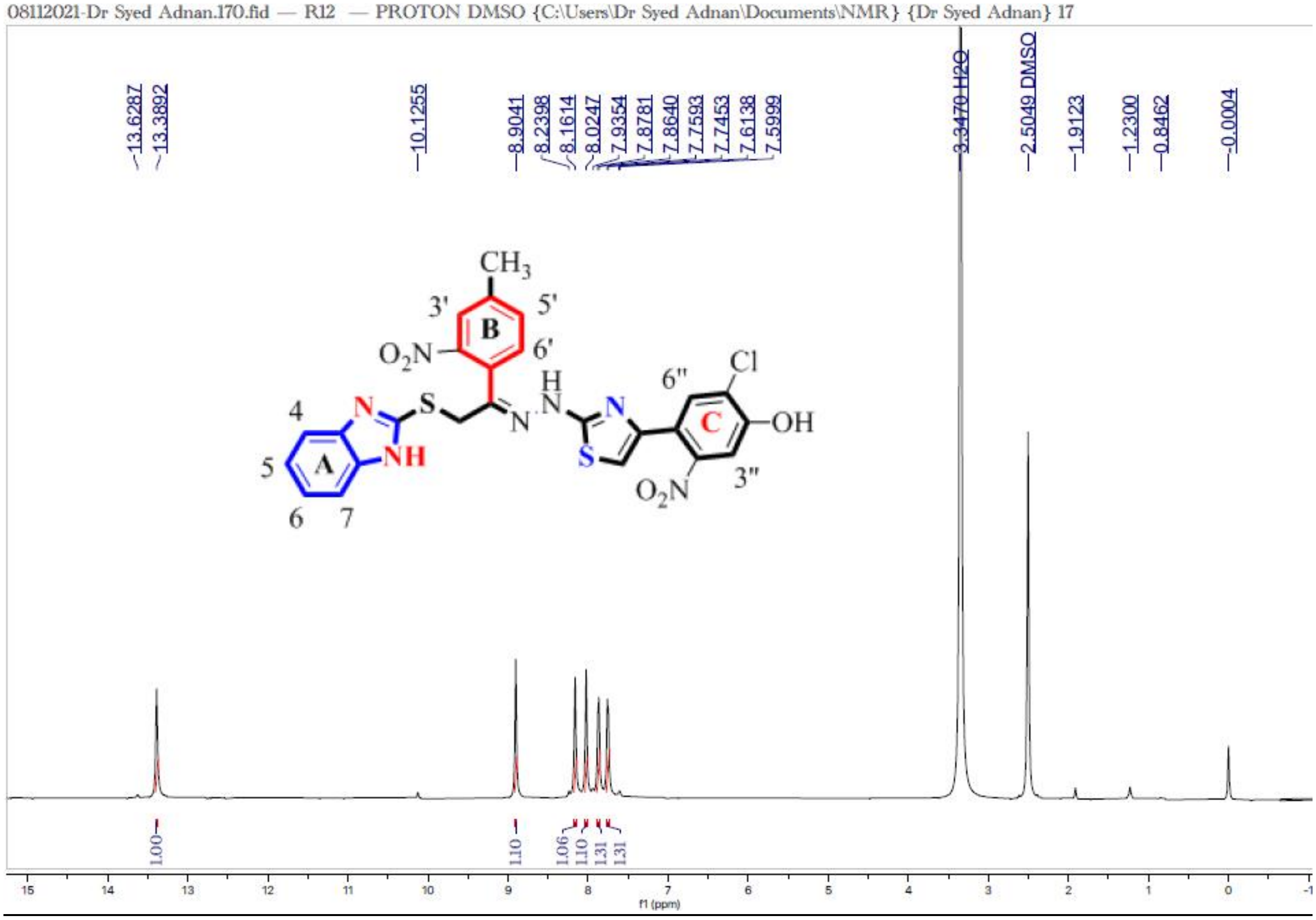


**Figure S12:** $^{1}H$-NMR of (E)-4-(2-(2-(2-((1H-benzo[d]imidazol-2-yl)thio)-1-(4-methyl-2-nitrophenyl)ethylidene)hydrazinyl)thiazol-4-yl)-2-chloro-5-nitrophenol (12)

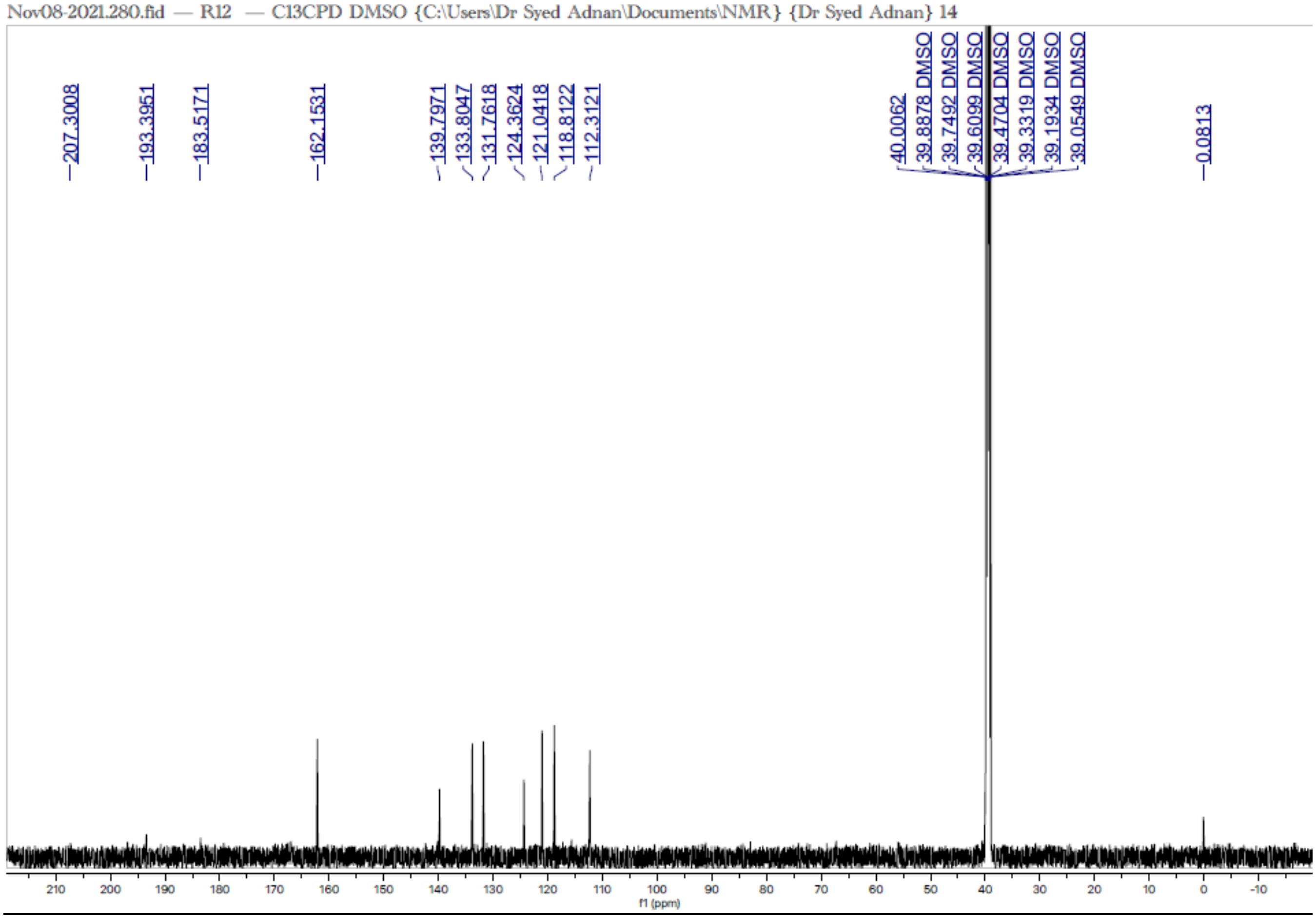


**Figure S13:** $^{13}$C-NMR of (E)-4-(2-(2-(2-((1H-benzo[d]imidazol-2-yl)thio)-1-(4-methyl-2-nitrophenyl)ethylidene)hydrazinyl)thiazol-4-yl)-2-chloro-5-nitrophenol (12)
